# CNNG: A Convolutional Neural Networks With Gated Recurrent Units for Autism Spectrum Disorder Classification

**DOI:** 10.3389/fnagi.2022.948704

**Published:** 2022-07-05

**Authors:** Wenjing Jiang, Shuaiqi Liu, Hong Zhang, Xiuming Sun, Shui-Hua Wang, Jie Zhao, Jingwen Yan

**Affiliations:** ^1^College of Electronic and Information Engineering, Hebei University, Baoding, China; ^2^Machine Vision Technological Innovation Center of Hebei, Baoding, China; ^3^School of Mathematics and Information Science, Zhangjiakou University, Zhangjiakou, China; ^4^School of Computer Science and Technology, Henan Polytechnic University, Jiaozuo, China; ^5^School of Engineering, Shantou University, Shantou, China

**Keywords:** ASD classification, CNNG, CNN, spatio-temporal features, ABIDE

## Abstract

As a neurodevelopmental disorder, autism spectrum disorder (ASD) severely affects the living conditions of patients and their families. Early diagnosis of ASD can enable the disease to be effectively intervened in the early stage of development. In this paper, we present an ASD classification network defined as CNNG by combining of convolutional neural network (CNN) and gate recurrent unit (GRU). First, CNNG extracts the 3D spatial features of functional magnetic resonance imaging (fMRI) data by using the convolutional layer of the 3D CNN. Second, CNNG extracts the temporal features by using the GRU and finally classifies them by using the Sigmoid function. The performance of CNNG was validated on the international public data—autism brain imaging data exchange (ABIDE) dataset. According to the experiments, CNNG can be highly effective in extracting the spatio-temporal features of fMRI and achieving a classification accuracy of 72.46%.

## Introduction

The neurodegenerative diseases such as autism spectrum disorder (ASD) have received increasing attention in recent years. ASD, also referred to as autism, is a common neurodevelopmental cognitive disorder in children, mostly related to genetic factors. Due to the unclear etiology of autism, lack of specific drug treatment and life-long incurable, the patient’s family needs to bear heavy psychological and economic pressure for a long time. ASD is characterized by complexity and heterogeneity. ASD mainly relies on the doctor’s diagnosis on the foundation of the Diagnostic and Statistical Manual of Mental Disorders. It is not only time-consuming but also highly subjective, which can easily lead to misdiagnosis. Therefore, the development of a fully automatic ASD diagnostic technology will alleviate the burden on doctors and be helpful to detect symptoms and obtain early intervention and treatment in childhood.

With the development of medical imaging, many functional neuroimaging techniques have been proposed to use in brain research, such as Electroencephalogram (EEG), magnetic resonance imaging (MRI), functional magnetic resonance imaging (fMRI), and so on (Laxer, 1997; Wu et al., 2001; Holdsworth and Bammer, 2008). fMRI has the advantages of non-invasiveness and high temporal and spatial resolution. fMRI can enable people to more intuitively understand the physiological and pathological functional activities of the brain. Therefore, fMRI is widely used in clinical and basic research in many fields such as neuroscience, cognitive science and psychology (Heuvel and Pol, 2010; Liu S. et al., 2019; Liu S. et al., 2020; Vakamudi et al., 2020). The fMRI which takes blood oxygenation level dependent (BOLD) signal imaging as the fundamental principle can be divided into task state and resting state in brain research. Task-fMRI means that fMRI data is collected by subjects under the specified task, such as staring at a certain color of a certain mark or moving a finger for a period of time. As a method of acquiring brain signals with the high spatial and temporal resolution, resting-state fMRI (rs-fMRI) requires subjects in a state of complete relaxation without accepting any specified or strenuous tasks. The acquisition method is simple and fast and is suitable for ASD patients, so it is widely used in ASD classification. As with most classification studies of neurological disorders, the data used in this paper were resting-state fMRI. Due to the lack of subtype data of ASD in the current public datasets, ASD classification studies are mainly aimed at dichotomizing ASD and typical controls (TC). We also aim to distinguish ASD and TC.

In recent years, with the advancements in computer technology and machine learning, artificial intelligence has been broadly applied in different industrial fields. Scholars are committed to using machine learning to process and analyze medical data. The processing based on medical data has received more and more attention from researchers. Brain neuroimaging has also gradually provided a new way for the classification research of brain neurological diseases. The study of fMRI-based ASD classification can be divided into two directions in terms of model composition: traditional machine learning and deep learning.

Scholars from various countries have proposed different ASD classification and identification methods with traditional machine learning. The main steps include feature extraction and classification. In 2015, Plitt et al. (2015) used three groups of regions of interest to generate three independent fMRI time-course correlation matrices for subjects. Then, the generated feature matrix is used for classification by combining linear kernel-based support vector machine (SVM), and the classification accuracy was 73.89% in 178 subjects. In 2020, Wang et al. (2020) put forward a multi-site adaption framework via low-rank representation decomposition to address the differences between multiple sites. The key idea is to establish a common low-rank representation for data from multiple sites. One site can be treated as the target domain and the rest as the source domain. So, each site can be mapped into a common space by using the low-rank representation. It can reduce the distribution difference of data at different sites by using the data of the target domain to linearly represent the data of the source domain. Finally, the proposed algorithm used a linear kernel-based SVM classifier for ASD classification, and its classification accuracy is 71.88% in 468 subjects. In 2020, Zhao et al. (2020) extracted the time-invariant features in the low-order or high-order dynamic functional connectivity network of fMRI data by using central moment. By integrating the traditional functional connectivity network, the low-order dynamic functional connectivity network and features were extracted from the high-order dynamic functional connectivity network, and a linear kernel-based SVM classifier was used to obtain up to 83.00% accuracy in 45 ASD patients and 47 TCs. In the same year, Karampasi et al. (2020) used the time series extracted by the CC200 atlas, demographic information, texture and divergence features of the BOLD signal as manually extracted features. Then, five feature selection algorithms such as recursive feature elimination with correlation bias reduction, local learning, infinite feature selection, minimum redundancy maximum correlation and Laplace score were used for feature selection. Finally, SVM based on linear kernel and Gaussian kernel, K-nearest neighbor classifier, linear discriminant analysis and random forest were used for ASD classification. Among them, the linear kernel-based SVM classifier achieved the highest classification accuracy of 72.5% among 871 subjects. Sun et al. (2021) first investigated the statistical differences among six resting-state networks. Then, they analyzed subjects with independent component analysis and applied an image-based meta-analysis to explore the consistency of spatial patterns across different sites. Finally, using these patterns as features, the results were predicted by an SVM classifier based on the Gaussian radial basis sum function. The six resting-state networks achieved classification accuracies of 66.10, 53.20, 59.70, 50.00, 75.80, and 88.70% in 295 subjects, respectively.

The process of feature selection in traditional machine learning algorithms is often accompanied by a certain degree of subjectivity. With the rapid progress of computer technology, classification algorithms based on deep learning have gained popularity. Deep learning-based methods can learn optimal classification strategies directly from raw data by using hierarchies of varying complexity. Compared with traditional machine learning methods, it has stronger classification and recognition capabilities. In 2018, Heinsfeld et al. (2018) used the CC200 functional atlas to segment the brain into 200 regions of interest (ROI) and calculated the Pearson correlation coefficient between each ROI to generate a functional connectivity matrix. Then, by removing the upper triangular and diagonal parts of the functional connectivity matrix, the remaining parts were spread into a one-dimensional vector to be used as classification features. Finally, two stacked denoising self-encode network with Softmax activation function was used for ASD classification, which obtained an accuracy of 70% in 1,035 subjects. In 2018, Xiao et al. (2018) divided the dataset of each subject into 30 independent components. Then, 20 key components were selected based on the maximum energy criterion for all bands. The array of 84 key features for all subjects was reshaped into a 3,400*84-dimensional key feature matrix. After performing normalization, the feature matrix was fed into a stacked autoencoder and the subjects were classified by using a Softmax classifier, which obtained an average classification accuracy of 87.21% in 84 subjects. In 2019, Rathore et al. (2019) obtained a classification accuracy of 69.2% in 1,035 subjects by using a simple 3-layer neural network with functional correlation and its topological features of EEG signals. In 2020, Thomas et al. (2020) trained a full 3D CNN containing only the average pooling layer and two convolutional layers, and the classification accuracy achieved 66% on 1,162 subjects. Niu et al. (2020) introduced a multichannel deep attention neural network for ASD classification, whose classification accuracy achieved 73.2% in 809 subjects. Li et al. (2020b) put forward an ASD classification algorithm on the basis of interpretable graph neural networks. In this algorithm, each graph convolution block contains a nodal convolution layer and a nodal pooling layer. This algorithm segmented brain images into 84 ROIs by using Desikan-Killiany mapping and constructed a functional connectivity matrix by using Pearson correlation coefficients. The functional connectivity matrix was fed into the proposed graph neural network for ASD classification, which obtained 79.7% classification accuracy in 118 subjects.

In 2019, Khosla et al. (2019) extracted ROI time series features by different atlases and further proposed an integrated learning strategy based on 3D CNN. The new network used the full-resolution 3D spatial structure of rs-fMRI data to fit a non-linear prediction model and obtained a classification result of 72.8%. Li et al. (2020a) presented an ASD classification algorithm by combining attention, long and short-term memory recurrent neural networks and self-encoder networks. This algorithm utilized functional connectivity as a feature and achieved 71.3% inter-site classification accuracy.

Functional magnetic resonance imaging images are an arrangement of a series of three-dimensional images obtained in a time series with a large number of data voxels. Most current methods used atlases to segment the brain into multiple regions of interest and construct a functional connectivity matrix as features. Then feature extraction methods were used to select some of the optimal features to input into a classifier for ASD classification. These algorithms did not fully exploit the spatio-temporal information of the source images. And they destroyed the temporal and spatial correlation of the original data. Therefore, we design an ASD classification algorithm based on 3D CNN and GRU. The representative high-level features of 3D images at each time point are gradually extracted by 3D convolutional neural networks. Then, the above spatial features at each time point are fed into GRU in series to analyze their temporal correlation information. Finally, a fully connected layer with a Sigmoid activation function is used to predict the category.

The main contributions of this paper are: (1) We combine the strengths of 3D CNN and GRU to construct a CNNG network. The CNNG network performs well in extracting the spatio-temporal features of fMRI data and hence obtains better ASD classification performance. (2) CNNG adopts intercepting time dimension, scaling brain image size as well as regularization and Dropout to prevent the overfitting phenomenon during model training. (3) We select the data of 871 subjects in the commonly used ABIDE database as the experimental data so that the trained model has better generalization ability for the diagnosis of ASD.

## The Proposed Algorithm

**FIGURE 1:**
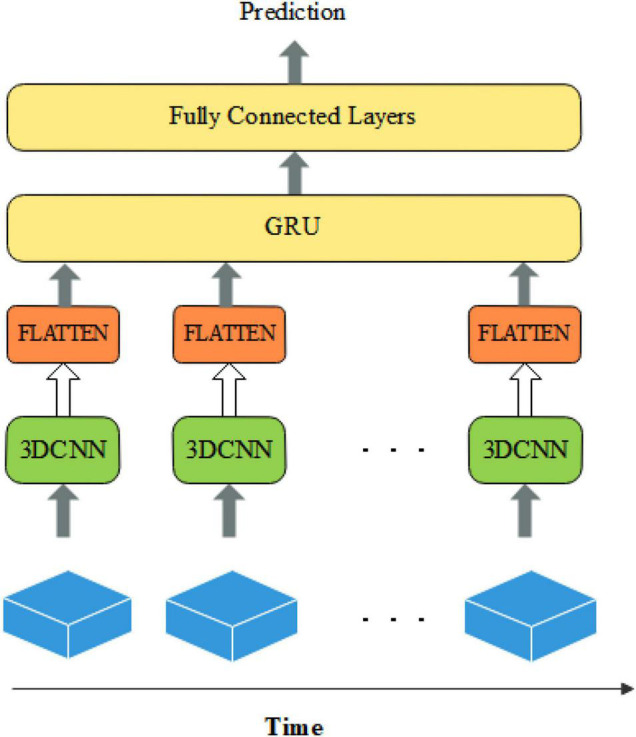
The structure of the CNNG model.

Some studies have been conducted for ASD classification by using CNN (Li et al., 2018; El-Gazzar et al., 2019). Because of the complexity and high dimensionality of fMRI images, only a few studies are using intact brain images directly as input data. Researchers have devoted themselves to reducing the input dimensionality by downscaling four-dimensional images into two-dimensional images or segmenting brain regions to construct functional connectivity matrices. And then the CNN networks or brain functional networks are constructed for classification. However, the above methods severely neglect the spatio-temporal information in fMRI data. Because the original fMRI data has high spatial and temporal dimensions, it will cause a serious overfitting phenomenon when the original fMRI data is the direct input of the network. Therefore, in this paper, the fMRI data are spatially reduced and intercepted with fixed temporal dimensions. For better results, we construct a deep learning classification model based on spatio-temporal features by combining it with a 3D convolutional neural network and gated recurrent unit, called the CNNG model. The CNNG model uses multiple 3D CNN networks with shared weights to extract the spatial features of brain images at each time point, and then uses the GRU to resolve the temporal information. The structure of the CNNG model is presented in Figure 1.

Figure 1 illustrates that the 3D spatial structure of fMRI at each time point is sent to 3D CNN for spatial feature extraction. The extracted spatial features are separately flattened and sent to GRU for temporal feature extraction. The last layer is the fully connected layers (FC) with the Sigmoid activation function, which predicted classification results. Each node of the layer in FC is attached to all nodes of the previous layer, and the features extracted in the previous layer are combined to output the prediction probability. Each part is described in detail below.

### Three-Dimensional Convolutional Neural Network

A convolutional neural network is a deep feed-forward neural network with local connectivity features and weight sharing. 3D convolution extends 2D convolution to 3D and extracts features of 3D data by 3D kernel convolution. Assuming that element $k_{\text{ij}}^{x_{0}\,y_{0}\,z_{0}}$ is the value at the position (*x*_0_,*y*_0_,*z*_0_) of the *j*-th feature map of the *i*-th layer, then the three-dimensional convolution can be expressed as:


(1) $$k_{\text{ij}}^{x_{0}\,y_{0}\,z_{0}}=\partial \left(b_{\text{ij}}+\sum_{c}\sum_{p=0}^{P_{i}-1}\sum_{q=0}^{Q_{i}-1}\sum_{r=0}^{R_{i}-1}w_{i\,j\,c}^{p\,q\,r}\,k_{\left(i-1\right)\,c}^{\left(x_{0}+p\right)\,\left(y_{0}+q\right)\,\left(z_{0}+r\right)}\right)$$

where ∂ is the activation function. *P*i, *Q*_i_, and *R*_*i*_ are the dimensional magnitudes of the three directions, respectively. $w_{\text{ijc}}^{p\,q\,r}$ is the value of the convolution kernel connecting the *c*-th feature map of the *i*−1-th layer with the j -th feature map of the i -th layer at the position (*p*,*q*,*r*). *b*_ij_ is bias.

Medical images contain two-dimensional, three-dimensional and four-dimensional images, etc. 3D convolution can extract spatial features of 3D images, which is increasingly used in medical image analysis. The fMRI contains data from 3D brain space images, so the 3D CNN is suitable for the 3D spatial feature extraction of fMRI. In CNN, large convolutional kernels can be replaced with repeated small convolutional kernels. The different sizes of the convolutional kernels bring the different sizes of the perceptual field. So, it is often used to replace one layer of large convolutional kernels with multiple layers of small convolutional kernels to reduce the number of parameters and computation while maintaining the same perceptual field. For example, it is very common to replace one layer of 5×5 convolutional kernels with two layers of 3×3 kernels, and to replace one layer of 7×7 kernels with three layers of 3×3 kernels.

**FIGURE 2:**
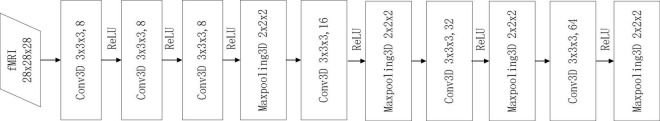
The structure of single-frame convolutional neural network (CNN).

The structure of the 3D CNN model used in this paper is presented in Figure 2. The input size of 3D CNN is 28×28×28, and it contains three convolutional layers. Each convolutional layer has 8 convolution kernels with the size of 3×3×3, and they are all connected with ReLU layers. The fourth layer is the maximum pooling layer with a step size of 2 and a kernel size of 2×2×2. The main purpose of the maximum pooling layer is to reduce the image size, prevent overfitting and reduce the running time. To extract more advanced features, three sets of repeated convolutional and pooling layers are added after the pooling layer. And the size of each convolutional kernel is 3×3×3. The number of filters in each convolutional kernel is 16, 32, and 64. The size of the pooling kernel after each convolutional layer is 2×2×2.

### Gated Circulation Unit

**FIGURE 3:**
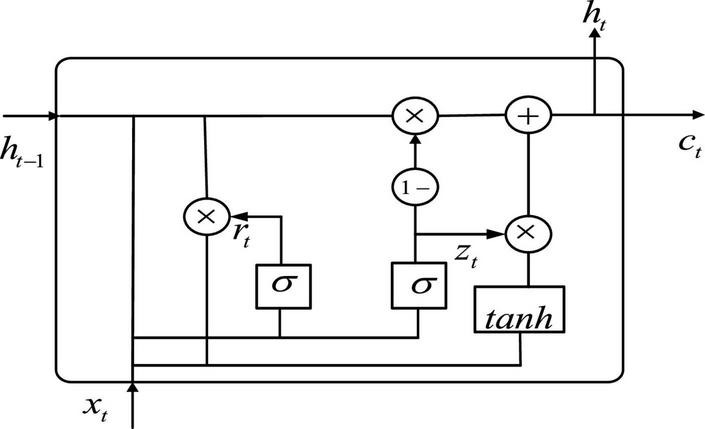
The structure of gate recurrent unit (GRU).

After extracting the fMRI spatial features by using 3D CNN, we use GRU to process the spatial features arranged along the time dimension after flattening. GRU is an improved version of long short-term memory (LSTM) presented by Cho et al. (2014), in which many ideas are borrowed from LSTM. LSTM has three inputs and three outputs, while the GRU has two inputs and two outputs (Xin et al., 2021; Liu S. et al., 2022). GRU can accelerate the training and enhance the network performance because of fewer parameters. The structure of GRU neurons is shown in Figure 3.

Let be the input of the GRU, and *c*_*t*_ is the output of the GRU. As can be seen from Figure 3, the expression of GRU is also slightly different from that of LSTM, with the following equation:


(2) $$z_{\text{t}}=\sigma \,\left(W_{\text{z}}\cdot \left[h_{t-1},x_{\text{t}}\right]\right)$$

(3) $$r_{\text{t}}=\sigma \,\left(W_{\text{r}}\cdot \left[h_{t-1},x_{\text{t}}\right]\right)$$

(4) $${\tilde{h}}_{\text{t}}=\tanh\,\left(W\cdot \left[r_{\text{t}}*h_{t-1},x_{\text{t}}\right]\right)$$

(5) $$h_{\text{t}}=\left(1-z_{\text{t}}\right)*h_{t-1}+z_{\text{t}}*{\tilde{h}}_{\text{t}}$$

where *z*_t_ denotes update gate. *r*_t_ denotes reset gate. ${\tilde{h}}_{\text{t}}$ denotes hidden unit. *h*_t_ is the current moment output. *W*_z_, *W*_r_, and *W* denote weights. *tanh* is the activation function.

In terms of operation, the GRU and LSTM work in a similar way. But the GRU unit uses a hidden state to combine the forgetting and input gates into a single update gate. It controls both how much information needs to be forgotten from the hidden layer of the previous moment and how much memory information from the hidden layer of the current moment is added. There is also a new “gate” in the GRU called the reset gate, which controls whether the computation of ${\tilde{h}}_{\text{t}}$ depends on state *h*_*t*−1_ at the previous moment. When, *r*_t_ = 0, ${\tilde{h}}_{\text{t}}$ is only related to the current input *x*_t_ and has nothing to do with the history state. When, *r*_t_ = 1, ${\tilde{h}}_{\text{t}}$ is related to *x*_t_ and *h*_*t*−1_. The advantage of GRU over LSTM is that there is less internal “gating” and fewer parameters than LSTM. GRU can achieve equivalent levels of performance, and it is easier to train, which can greatly increase training efficiency. Therefore, we use GRU for feature extraction in the time dimension to obtain better ASD classification results.

### Model Training

The proposed model extracts the spatial features before the temporal analysis. The specific parameter settings of the single-frame CNN model are given in Table 1. We first use two repeated three-dimensional convolutions with the size of 3×3×3 to extract the low-level features. Then, we use repeated pooling and convolution to extract the high-level features. And the repeated two-layer convolution is replaced by a single convolutional layer with a kernel size of 3×3×3, which is reduce the number of parameters. The extracted spatial features are flattened and input into a GRU with 32 neurons. Finally, the predicted values are output by a fully connected layer with a Sigmoid activation function.

**TABLE 1:** Structure of single-frame 3D convolutional neural network (CNN) model.

Layer	Type	Output size	Filter	Core size
1	Conv3D	28× 28× 28	8	3× 3× 3
2	Conv3D	28× 28× 28	8	3× 3× 3
3	Conv3D	28× 28× 28	8	3× 3× 3
4	MaxPooling3D	14× 14× 14	8	2× 2× 2
5	Conv3D	14× 14× 14	16	3× 3× 3
6	MaxPooling3D	7× 7× 7	16	2× 2× 2
7	Conv3D	7× 7× 7	32	3× 3× 3
8	MaxPooling3D	4× 4× 4	32	2× 2× 2
9	Conv3D	4× 4× 4	64	3× 3× 3
10	MaxPooling3D	2× 2× 2	64	2× 2× 2

The adaptive moment estimation (Adam) optimization algorithm is used for model optimization in the network training. The loss function is the cross-entropy loss function. The input batch size is set to 1, and the learning rate is 0.00001. Dropout means that in the training process of the network, neural network units are randomly discarded from the network according to a certain probability. To avoid overfitting of the proposed model, the values of dropout and recurrent_dropout of the parameters in GRU are set to 0.3. The two-parameter regularization is carried out in the Dense layer with the parameter 0.00001.

## Data Preprocessing

The rs-fMRI data used in this paper are from the international publicly available Autism Brain Imaging Data Sharing Project dataset. The dataset brings together 1,112 subjects (539 ASD patients and 573 TCs) from 17 sites around worldwide, including: California Institute of Technology (Caltech), Carnegie Mellon University (CMU), Kennedy Krieger Institute (KKI), Ludwig Maximilians University Munich (MaxMun), New York University Langone Medical Center (NYU), Olin Institute of Living at Hartford Hospital (Olin), Oregon Health and Oregon Health and Science University (OHSU), San Diego State University (SDSU), Social Brain Lab (SBL), Stanford University (Stanford), Trinity Centre for Health Sciences (Trinity), University of California, Los Angeles (UCLA), University of Leuven (Leuven), University of Michigan (UM), University of Pittsburgh School of Medicine (Pitt), University of Utah School of Medicine (USM), and Yale Child Study Center (Yale). The corresponding sites and the sizes of samples are shown in Table 2, and all the data can be downloaded from the official website from ABIDE I (2022). The database includes rs-fMRI, structural MRI, and extensive phenotypic information for each subject. In this paper, the subjects with missing partial information were excluded. A final dataset of 871 subjects, including 403 ASD patients and 468 TCs, was obtained by removing the samples with incomplete brain coverage, high motion peaks, ghosting and other scanner artifacts.

**TABLE 2:** Names of the 17 sites and their sample sizes.

Serial number	Sites	ASD	TC	Total subjects
1	Caltech	19	19	38
2	CMU	14	13	27
3	KKI	22	33	55
4	Leuven	29	35	64
5	MaxMun	24	33	57
6	NYU	79	105	184
7	OHSU	13	15	28
8	Olin	20	16	36
9	Pitt	30	27	57
10	SBL	15	15	30
11	SDSU	14	22	36
12	Stanford	20	20	40
13	Trinity	24	25	49
14	UCLA	62	47	109
15	UM	68	77	145
16	USM	58	43	101
17	Yale	28	28	56

During fMRI acquisition, a lot of noise is generated, so preprocessing is required before use. The preprocessing method used in this paper is the configurable pipeline for the analysis of connectomes (CPAC), and the specific processing steps are as follows:

(1) Time slice correction. There is a time difference in the acquisition of fMRI images. To ensure the accuracy of the images, 3dTshift of functional neuroimaging analysis was used to correct the time slices.

(2) Head movement correction. When collecting data, it is impossible to guarantee that the subject does not move at all. Some slight movements can lead to huge data differences, so head movement correction is needed.

(3) Alignment. The skewed functional or structural image is adjusted to the vicinity of the spatial standard position, so that the subsequent processing algorithm can quickly find the optimal value and ensure a higher quality alignment.

(4) Numerical normalization. The 4D fMRI images were globally normalized with the global mean value equal to 1,000.

(5) Interference signal regression. The Friston 24-parameter model regression was used to eliminate the head movement effect of the functional image after alignment. To reduce the effect of respiration and heartbeat, the regression was done. Regression is also used to remove low-frequency drift generated by the long machine operation (Wang, 2020).

(6) Filtering. To reduce the influence of noise such as breathing and heartbeat and remove the low-frequency drift and the high-frequency noise, the low-frequency signal in the range of 0.01–0.1 Hz is selected. This frequency band can reflect the individual’s spontaneous neural activity and has certain biological significance (Lu et al., 2007).

(7) Spatial normalization. In general, the size of the human brain varies. In order to unify the standard, the image space is normalized to the template space of the Montreal Neurological Institute with a resolution of 3×3×3*mm*^3^.

In the ABIDE dataset, the dimensions of the 3D spatial brain images were consistent for each site. While the temporal dimensions varied, the OHSU site had the least temporal dimension of 78, and the CMU site had the highest temporal dimension of 316. Since the model requires a fixed input size, the fMRI data is preprocessed before being fed into the network. Specifically, the fMRI of the first ten time points was removed in the time dimension, and 32 consecutive frames of 3D brain images were taken from the eleventh frame. Spatially, the spatial dimension of each image (61, 73, 61) is downsampled to (28, 28, 28). After the processing of temporal and spatial dimensions, the size of the obtained fMRI data is (28, 28, 28, 32). This ensures the same model input and preserves the spatio-temporal characteristics of fMRI. The selection of the time input size is discussed in detail in the experimental results analysis section.

## Experimental Results and Analysis

The experiments in this paper are based on the Tensenflow 1.0 platform. The environment is the Ubuntu18.4 operating system. The hardware is a server with 32G memory, Intel(R) Xeon(R) CPU E5-2667 processor and NVIDIA Tesla K40c.

As we all know, in the field of deep learning, it is very important to divide the training set and test set reasonably. For the traditional machine learning stage (the size of data set is less than 10,000), the general allocation ratio is that the ratio of training set to test set is 7:3 or 8:2. Try to keep the distribution of training set and test set consistent. For verifying the validity of CNNG and retaining as much training data as possible, the data is categorized into a training set and a test set in a ratio of 8:2.

In binary classification studies, Accuracy, Sensitivity and Specificity are commonly used indicators, which can be expressed as:


(6) $$A\,c\,c\,u\,r\,a\,c\,y=\frac{T\,P+T\,N}{T\,P+F\,P+T\,N+F\,N}$$

(7) $$S\,e\,n\,s\,i\,t\,i\,v\,i\,t\,y=\frac{T\,P}{T\,P+F\,N}$$

(8) $$S\,p\,e\,c\,i\,f\,i\,c\,i\,t\,y=\frac{T\,N}{T\,N+F\,P}$$

In this experiment, the label for ASD patients is “1,” and the label for TC is “0.” The above equation True Positive (TP) indicates the number of samples with label “1” predicted to the number of samples with label “1.” False Positive (FP) indicates the number of samples that predict a label of “0” to a label of n“1” True Negative (TN) indicates the number of samples with the label “0” predicted to the number of samples with the label “0.” False Negative (FN) indicates the number of samples with the label “1” predicted as the label “0.” TP+FP+TN+FN is the total number of samples. TP+FN is the total number of samples with the true label “1”. TP+FP is the total number of samples with the prediction label “1,” including both correct and incorrect predictions. FP+TN represents the total number of samples with the true label “0.” TN+FN represents the total number of samples with the prediction label “0,” including both correct and incorrect predictions.

It can be seen from the above description that the sensitivity reflects the ability of ASD patients to be correctly distinguished. The higher the sensitivity means the higher the probability that a patient with ASD will be correctly diagnosed. The specificity reflects the effect of TC subjects being correctly classified. The accuracy reflects the overall classification ability. The higher the accuracy, the greater the value for practical medical diagnosis applications.

### Ablation Experiments

#### Effects of Different Convolution Kernel Sizes

For the purpose of obtaining the optimal model, we select the number of convolution layers and the size of the convolution kernel by comparison experiments. First, the number of convolution layers and the kernel size before the first pooling layer are determined. The experiments were conducted by using convolution kernels with a size of 5×5×5 and 7×7×7 as well as replacing them with repeated small convolution kernels. The result is listed in Table 3. From Table 3, it is clear that the repeated small convolutional kernels have better classification results than the corresponding large convolutional kernels. For example, the superposition of three convolution kernels with a size of 3×3×3 achieves a maximum accuracy of 72.46%. It is about 2% higher than the accuracy of the model with the corresponding convolutional kernel size of 7×7×7. Similarly, when two convolution kernels with a size of 3×3×3 are superimposed, the accuracy rate is 70.04%. While the classification accuracy of the model with a convolution kernel size of 5×5×5 is only 67.63%. The above results show that the superposition of small convolutional kernels has the advantages of a small number of parameters and the low computational complexity. It is clear that the classification effect of only one convolution layer with a convolution kernel size of 3×3×3 is not ideal. It may be due to the fact that the receptive field is too small to effectively extract the features.

**TABLE 3:** Classification performance of different convolutional kernel sizes.

Kernel sizes (number of layers)	Accuracy	Sensitivity	Specificity
7 x 7x 7 (1)	70.53%	64.15%	77.23%
5 x 5 x 5 (1)	67.63%	62.37%	72.64%
3 x 3 x 3 (3)	**72.46%**	**65.35%**	**79.25**%
3 x 3 x 3 (2)	70.04%	65.09%	75.25%
3 x 3 x 3 (1)	62.32%	59.80%	66.04%

*The bold values in this table represent the optimal values of accuracy, specificity and sensitivity.*

#### Comparison of Long Short-Term Memory Module and Gate Recurrent Unit Module

Since LSTM and GRU are the variations of RNN, both are widely used in temporal information extraction. The classification results based on temporal feature extraction selection by LSTM and GRU, respectively, are shown in Table 4. When GRU is replaced by LSTM in the proposed method, the classification accuracy is 68.60%, the sensitivity is 56.60%, and the specificity is 81.19%. The accuracy is significantly lower compared to GRU, so we use GRU for temporal feature extraction.

**TABLE 4:** Performance of different temporal feature extraction modules.

Time feature extraction module	Accuracy	Sensitivity	Specificity
LSTM	68.60%	56.60%	**81.19%**
GRU	**72.46%**	**65.35%**	79.25%

*The bold values in this table represent the optimal values of accuracy, specificity and sensitivity.*

#### Effects of Different Time Dimensions

The selection of the number of fMRI time points has an important influence on the model training. In the time dimension, 8, 16, 32, and 48 frames of fMRI images are used for experiments in this paper. Table 5 presents the classification effects.

**TABLE 5:** Classification performance of different time interceptions.

Time dimension	Accuracy	Sensitivity	Specificity
8	63.74%	59.33%	67.32%
16	69.08%	63.46%	72.12%
32	**72.46%**	**65.35%**	**79.25%**
48	69.57%	62.37%	76.42%

*The bold values in this table represent the optimal values of accuracy, specificity and sensitivity.*

According to Table 5, the classification accuracy improves when the temporal dimension increases. However, it starts to decrease when the temporal dimension is 48. Specifically, when the time dimension is taken as eight, the classification accuracy is low. This may be mainly due to the short time resulting in the short feature vector extracted by the tandem CNN, which cannot extract the temporal features effectively. And when the time dimension is 48, the number of parameters and computation of model training increases, which may easily lead to the phenomenon of overfitting. So, in the proposed algorithm, we finally choose the data of 32-time points, which can archive the best classification effect.

#### Effects of Different Numbers of Gate Recurrent Units

The selection of GRU has experimented in the previous section, and the number of GRU units also determines the performance of CNNG. So, we set the number of GRU units to 16, 32 and 48 for experimental analysis. From Table 6, the accuracy, sensitivity and specificity are lower when the number of GRU units is too small or too lager. This is because when the number of GRU units is less than 32, the proposed model is limited by the number of units and is not sufficient to fully express the information contained in the temporal dimension of the fMRI data. And when the number of units increases to 48, the classification performance shows different degrees of degradation due to overfitting because the parameters of the units are too redundant. Therefore, the model performance is optimal when the number of GRU units is taken as 32.

**TABLE 6:** Classification performance with different numbers of gate recurrent unit (GRU) units.

Number of GRU units	Accuracy	Sensitivity	Specificity
16	71.50%	64.15%	77.28%
32	**72.46%**	**65.35%**	**79.25%**
48	71.01%	62.38%	74.26%

*The bold values in this table represent the optimal values of accuracy, specificity and sensitivity.*

### Comparison With Traditional Machine Learning Algorithms

For verifying the validity of CNNG, we compare it with the ASD classification algorithm by using traditional machine learning. The comparison algorithms are: (1) ASD classification algorithm by using graph Fourier transform (GFT) and support vector machine proposed by Brahim and Farrugia (2020), which is abbreviated as RBF-SVC; (2) An ASD classification algorithm based on functional connectivity networks and recursive-clustering elimination support vector machine proposed by Chaitra et al. (2020), which is abbreviated as RCE-SVM; (3) A hybrid ASD classification algorithm by combining different brain segmentation definitions, functional connectivity matrix construction methods and feature extraction methods proposed by Graa and Silva (2021), which is abbreviated as HFR; (4) The ASD classification algorithm proposed by Abraham et al. (2016) based on CC400 brain atlas and support vector machine, which is abbreviated as C-SVC; (5) The ASD classification algorithm on the basis of functional connectivity and ridge regression classifier proposed by Yang et al. (2019), which is abbreviated as FCR. The test results of the compared algorithm used in this paper are all from the test results of the code offered by the author in the corresponding reference. The test set used in this paper comes from 17 different sites, so the final metrics obtained are the average accuracy, average sensitivity and specificity. Table 7 presents the performance of CNNG and the comparison algorithm on the test set.

**TABLE 7:** Classification performance of traditional machine learning algorithms and CNNG.

Classification	Accuracy	Sensitivity	Specificity
RBF-SVC	66.70%	62.35%	72.35%
RCE-SVM	67.30%	64.5%	70.10%
HFR	71.10%	67.00%	75.00%
C-SVC	67.00%	53.20%	78.30%
FCR	71.98%	70.89%	71.53%
CNNG	**72.46%**	**71.35%**	**79.25%**

*The bold values in this table represent the optimal values of accuracy, specificity and sensitivity.*

As presented in Table 7, the accuracy, sensitivity and specificity of the CNNG model in 871 subjects reached 72.46, 71.35, and 79.25%, respectively. The accuracy is obviously higher than other traditional machine learning algorithms. When classifying ASD, many traditional machine learning algorithms need to divide the brain into multiple regions of interest, which is treated as a node for subsequent feature selection or calculation. This process obviously loses fMRI spatial information of the data. After the original image is preprocessed, the CNNG model directly extracts and classifies features through the model, which fully exploits the spatiotemporal information of fMRI data, thereby extracting more discriminative features and further enhancing the classification capability of the algorithm. In addition, the manual features extracted by the fixed computational feature algorithm are sensitive to noise, scanning equipment and parameters, and make a big influence on the overall classification capability of traditional machine learning algorithms.

### Comparison With Deep Learning Algorithms

We also carry out a comparison between CNNG and the deep learning-based ASD classification algorithm. The comparison algorithms are: (1) The ASD classification algorithm based on convolutional neural network and multilayer perceptron presented by Sherkatghanad et al. (2020), which is abbreviated as CNN-MLP; (2) The ASD classification algorithm based on functional connection network, extreme random tree and support vector machine proposed by Liu Y. et al. (2020), which is abbreviated as SVC; (3) The ASD classification algorithm based on joint representation learning deep multimodal model proposed by Eslami et al. (2019), which is abbreviated as DiagNet; (4) The ASD classification algorithm based on the hierarchical graph convolutional neural network framework introduced by Hao et al. (2020), which is abbreviated as HI-GCN; (5) The ASD classification algorithm based on graph attention network proposed by Hu et al. (2021), which is abbreviated as GAT. The test results of the comparison algorithm used in this paper are all from the test results of the code offered by the author in the corresponding reference. The test set used in this paper comes from 17 different sites, so the final metrics obtained are the average accuracy, average sensitivity and specificity. Table 8 presents the results of CNNG and the comparison algorithm on the test set.

**TABLE 8:** Classification performance of deep learning algorithms and CNNG.

Classification	Accuracy	Sensitivity	Specificity
CNN-MLP	70.22%	62.35%	72.35%
SVC	71.10%	67.00%	75.00%
DiagNet	70.30%	68.03%	72.20%
HI-GCN	67.20%	65.90%	68.40%
GAT	68.02%	74.06%	62.26%
CNNG	**72.46%**	**74.35%**	**79.25%**

*The bold values in this table represent the optimal values of accuracy, specificity and sensitivity.*

**FIGURE 4:**
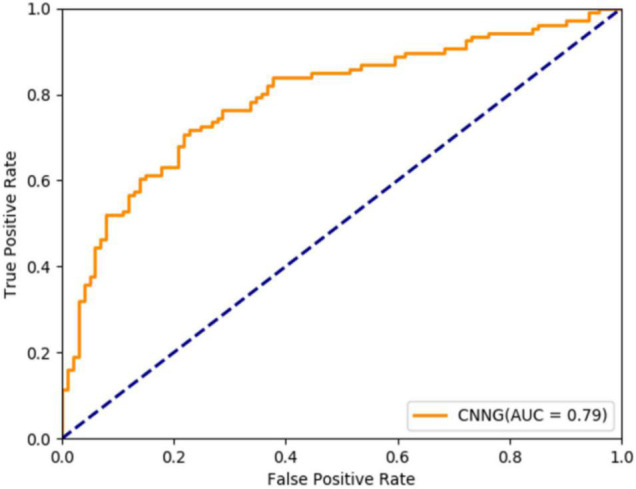
Receiver operating characteristic (ROC) curve of CNNG model.

Table 8 shows that the proposed algorithm obtains an accuracy of 72.46% in the experiment of 871 subjects, which is 5.26% higher than that of HI-GCN, 4.44% higher than that of GAT, and 2.44% higher than that of CNN. It is also a certain improvement compared to SVC and DiagNet. The proposed algorithm also obtains a specificity of 79.25% and a sensitivity of 74.35%. All the results reveal that the overall performance of CNNG is superior to the other deep learning algorithms, which suggests that directly extracting spatio-temporal features from 4D fMRI data for classification has better results for ASD classification than just by using 2D or 3D fMRI data or functional connectivity. For further evaluating the performance of CNNG, the receiver operating characteristic (ROC) curve and the area under the curve (AUC) values are plotted in Figure 4. In Figure 4, the horizontal coordinate represents the false positive rate (FPR) and the vertical coordinate represents the true positive rate (TPR). The ROC curve reflects the trend of the TPR and the FPR. The closer the area is to 1, the stronger the recognition ability is. Among the above proposed deep learning algorithms, the values of AUC for CNN-MLP, DiagNet, HI-GCN, and GAT are 0.7486, 0.764, 0.745, and 0.7358, respectively. As shown in Figure 4, the AUC value of the proposed algorithm is 0.79, which is 5.42% higher compared to GAT. This is an improvement of 4.5% compared to HI-GCN. Compared to CNN-MLP, the ACU of CNNG is improved by 4.14%. There is also a magnitude improvement compared to DiagNet. These data indicate that CNNG performs well for classification.

## Conclusion

In this paper, we put forward a deep learning model—CNNG, which can fully exploit the spatio-temporal information in fMRI data to avoid excessive dimensionality reduction and missing information caused by using the manual features for classification. CNNG is mainly composed of 3D CNN and GRU. In CNNG, spatial feature extraction is extracted by using 3D CNN, and then GRU is used to analyze temporal information. The validity of the CNNG model is proved by comparing it with the algorithms based on traditional machine learning and the algorithms based on deep learning. The experimental results indicate that CNNG performs better than other algorithms for ASD classification. CNNG can extract fMRI data features from the perspective of spatio-temporal convolution, which has some clinical value for the early diagnosis of ASD. At present, the sensitivity of the proposed algorithm does not obtain a large improvement, and next, we will optimize the algorithm for better ASD classification.

## Data Availability Statement

Publicly available datasets were analyzed in this study. This data can be found here: Autism Brain Imaging Data Exchange (ABIDE) dataset.

## Ethics Statement

The studies involving human participants were reviewed and approved by Autism Brain Imaging Data Exchange (ABIDE) dataset. The patients/participants provided their written informed consent to participate in this study.

## Author Contributions

HZ performed the computer simulations. SL, XS, and JZ analyzed the data. SL and WJ wrote the original draft. XS and S-HW revised and edited the manuscript. JY polished the manuscript. All authors confirmed the submitted version.

## Conflict of Interest

The authors declare that the research was conducted in the absence of any commercial or financial relationships that could be construed as a potential conflict of interest.

## Publisher’s Note

All claims expressed in this article are solely those of the authors and do not necessarily represent those of their affiliated organizations, or those of the publisher, the editors and the reviewers. Any product that may be evaluated in this article, or claim that may be made by its manufacturer, is not guaranteed or endorsed by the publisher.
